# Lost in the crowd? Using eye-tracking to investigate the effect of complexity on attribute non-attendance in discrete choice experiments

**DOI:** 10.1186/s12911-016-0251-1

**Published:** 2016-02-03

**Authors:** Jean Spinks, Duncan Mortimer

**Affiliations:** 1 Centre for Health Economics, Monash University, Melbourne, Australia; 2 Centre for Applied Health Economics, Menzies Health Institute (Queensland), Griffith University (Logan Campus - LO3 2.15), University Drive, Meadowbrook, Brisbane, QLD 4131 Australia

**Keywords:** Attribute non-attendance, Complexity, Information processing, Eye tracking, Complementary medicine, Labelling

## Abstract

**Background:**

The provision of additional information is often assumed to improve consumption decisions, allowing consumers to more accurately weigh the costs and benefits of alternatives. However, increasing the complexity of decision problems may prompt changes in information processing. This is particularly relevant for experimental methods such as discrete choice experiments (DCEs) where the researcher can manipulate the complexity of the decision problem. The primary aims of this study are (i) to test whether consumers actually process additional information in an already complex decision problem, and (ii) consider the implications of any such ‘complexity-driven’ changes in information processing for design and analysis of DCEs.

**Methods:**

A discrete choice experiment (DCE) is used to simulate a complex decision problem; here, the choice between complementary and conventional medicine for different health conditions. Eye-tracking technology is used to capture the number of times and the duration that a participant looks at any part of a computer screen during completion of DCE choice sets. From this we can analyse what has become known in the DCE literature as ‘attribute non-attendance’ (ANA). Using data from 32 participants, we model the likelihood of ANA as a function of choice set complexity and respondent characteristics using fixed and random effects models to account for repeated choice set completion. We also model whether participants are consistent with regard to which characteristics (attributes) they consider across choice sets.

**Results:**

We find that complexity is the strongest predictor of ANA when other possible influences, such as time pressure, ordering effects, survey specific effects and socio-demographic variables (including proxies for prior experience with the decision problem) are considered. We also find that most participants do not apply a consistent information processing strategy across choice sets.

**Conclusions:**

Eye-tracking technology shows promise as a way of obtaining additional information from consumer research, improving DCE design, and informing the design of policy measures. With regards to DCE design, results from the present study suggest that eye-tracking data can identify the point at which adding complexity (and realism) to DCE choice scenarios becomes self-defeating due to unacceptable increases in ANA. Eye-tracking data therefore has clear application in the construction of guidelines for DCE design and during piloting of DCE choice scenarios. With regards to design of policy measures such as labelling requirements for CAM and conventional medicines, the provision of additional information has the potential to make difficult decisions even harder and may not have the desired effect on decision-making.

**Electronic supplementary material:**

The online version of this article (doi:10.1186/s12911-016-0251-1) contains supplementary material, which is available to authorized users.

## Background

The use of discrete choice experiments (DCEs) in health care has increased dramatically over the past decade [1–4]. Arising from the disciplines of psychology and economics, the theoretical basis for DCEs can be found in random utility theory (RUT), developed by McFadden [5] and later Hanemann [6]. There is increasing evidence suggesting that decision making of the type emulated by DCEs is prone to diversions from the underlying theory [7, 8], which assumes that consumers are both fully informed and make ‘rational’ (optimising) decisions.

Mainstream economic models typically assume that consumption choices can be improved simply by providing people with more and better information. There are, however, many situations where this assumption may not hold due to limits on information-processing capacity. For very complex problems, consumers may be boundedly (rather than fully) rational [9, 10] and there is evidence to suggest that consumers attempting to evaluate all available information and all available options are increasingly likely to make mistakes through this process [11]. Many consumers will instead employ a ‘satisficing’ [12] or ‘fast and frugal’ [13] heuristic whereby the mental task of calculating the cost and consequences of all possible options is overwhelming; taking mental short-cuts to make decisions easier [14]. Recent findings from behavioural economics confirm that increases in the complexity of decision tasks may paralyse decision-making [15], although others argue that it is the nature of the information that is important, rather than the absolute amount [16]. One area of recent research activity focuses on so-called ‘attribute non-attendance’ (ANA) [17, 18] which in simple terms means that individuals may either ignore or attach threshold values to certain product characteristics before considering them. In the presence of ANA, DCE data may not characterise the preferences of affected individuals and standard approaches to analysis may produce biased estimates of the relative importance of product attributes [19].

Empirically, two main methods have been employed to assess the existence and extent of ANA - (i) using qualitative methods such as think-aloud protocols alongside stated-preference surveys [20], in-depth interviews and other supplementary questioning [21] to directly question the respondent about their cognitive processing strategy in answering stated-preference surveys; and (ii) using quantitative models that allow the researcher some latitude for inference, such as latent-class models, to analyse stated-preference data [22–25]. From this growing literature, it does appear that ANA may in fact be important when assessing the validity of stated-preference studies [17, 26] and that modelled coefficients should be adjusted accordingly. However, there are limitations when using both methods to reliably assess the presence and extent of any ANA.

Eye-tracking technology provides a novel alternative capable of directly measuring ANA without interfering with the decision making process or being constrained by computational limitations. First described in the 1970s [27], eye-tracking technology allows the researcher to record where and for how long a respondent to a computer-based survey focuses their eyes. This means that researchers can assess if, and for how long, each attribute or choice is focused relative to all else, including the sequence of focusing. If this information can be meaningfully interpreted, it may be used to determine whether attribute non-attendance is directly evident, whether systematic departures from the underlying theory can be identified, and ultimately, to inform how the predictive power of choice models can be improved to account for violations of the underlying assumptions.

A small number of research groups have begun exploring use of eye-tracking technology to understand decision-making. For example, Rasch et al. [28] use a combination of eye-tracking and facial electromyography to study affect in DCE decision making as it relates to marketing decisions; Arieli et al. [29] looks at decision-making under conditions of uncertainty (not in a DCE context); and, most relevant to the discussion here, Balcombe et al. [30] studied ANA within a DCE context as it relates to food nutrition labels. All of these studies found evidence of deviation from the underlying assumptions, acknowledging that work in this area is just beginning and there is still much to learn about the extent and effect that such deviations might have on the predictive ability of choice modelling.

Here, we make use of eye-tracking in simulated consumption decisions using a DCE framework to understand the process of consumer decision making in a complex, yet familiar, health environment – the purchase of medicine to treat a minor ailment. To begin with, we assess the presence of ANA under varying conditions of complexity and framing (different ailments). We then look at whether particular product attributes are more prone to ANA than others. Next, we focus on the potential determinants of any ANA found. As suggested by Lagarde [25], information processing is “…likely to be influenced by the decision problem itself (e.g. its complexity), respondent specific characteristics (e.g. familiarity to the choice task, cognitive skills) and the broader context in which the choice task is taken (e.g. time pressure)”. Using this framework, we aim to model ANA as a function of these influences in an attempt to identify their relative importance. Finally, we test the assumption made in previous work in this area [23] that respondents are consistent with their information processing rules, that is, “the decision on which attributes to consider does not change over the choices made by the same respondent” (page 205).

## Methods

### Study context

Our data was collected alongside the pilot study of a DCE which tests the effect of providing consumers with additional information in the form of (i) regulatory statements; and /or (ii) summary information in the form of a ‘traffic light’ logo, on the label of both ‘complementary’ (natural) or ‘conventional’ (pharmaceutical) medicines. Two different decision ‘frames’ were tested in the form of two common ailments: sleep problems and joint pain [see Additional file 1: Figures A1 and A2]. The design of the main DCE aimed to address a real and current policy issue – whether consumers make better (or different) medicine purchasing decisions if compulsory labelling changes are implemented in an attempt to simplify the purchasing decision [31].

Different wordings of the proposed regulatory statement have appeared in the literature or the media [32–34] [see Additional file 1: Figure A1 for descriptions]. Thus, we aim to test the potential effect on information processing of adding such statements to the already large amount of information that must be processed by consumers. As an alternative to regulatory statements, we also investigate the addition of a traffic-light advisory system, similar to what is being used on many foods [35, 36], as a way of highlighting key information for consumers [see Additional file 1: Figure A2].

### Participants

As geographical proximity was required (the eye-trackers were located at Monash University, Melbourne), a local recruitment strategy was necessary. Members of the University Staff (both academic and administrative) were invited to participate through a regular university e-newsletter. We focused on staff rather than undergraduate students (although PhD students were allowed to participate) so as to gain a more representative group in terms of demographics such as age and health status. Ethics approval was granted by Monash University [CF11/2535 – 2011001482] and all participants provided written informed consent.

### Choice scenarios

A DCE is one way of simulating the consumption choice and estimating how consumers may behave when characteristics (attributes) of the different choices (alternatives) are altered. By accounting statistically for the different levels of attributes presented, researchers can estimate the relative contributions of the different attributes towards the chosen alternative. The intention of the present study is not, however, to estimate part-worth utilities and we were not constrained by considerations of efficiency or orthogonality that would motivate use of a formal experimental design when constructing choice scenarios. In the present study, we manually constructed choice scenarios (described below) to simulate the effect of complexity on decision-making and to allow observation and recollection of decision-processes using eye-tracking and semi-structured interviews.^1^ Methods and results from the larger DCE using an experimental design (permitting efficient estimation of part-worth utilities) are reported elsewhere [31].

The online survey included eight choice scenarios per respondent, split equally across the two health conditions. To test the influence of complexity of the choice scenario (and cognitive burden), we allowed the number of attributes presented in choice scenarios to vary from three to eight (see Table 2). Half the participants were presented with an increasing number of attributes (increasing complexity); the other half was shown a decreasing number of attributes (decreasing complexity). In an attempt to minimise unthinking / mechanical choice, levels of attributes were varied across choice scenarios to obtain as much attribute balance as possible within the constraints of the study design.

Participants in the present study were asked to consider one of two scenarios – both of which describe mild health conditions (insomnia or joint pain) for which a range of self-care options are available. These two conditions were chosen due to their prevalence in the general population as well as the availability of both complementary and conventional medicines for self-selection and treatment. Within each condition, participants were asked to choose between three alternatives - a conventional medicine, a complementary medicine and ‘neither of these’ (opt out option).

This study forms part of a larger, multi-disciplinary project focused on complementary and alternative medicine (CAM) use in people with chronic illness. The identification of attributes and levels for inclusion in the DCE choice scenarios therefore drew on qualitative work completed as part of the broader project, as well as a survey in the target population (*N* = 2,915) describing motivations for and use of CAM alongside conventional medicine [37–39]. A summary of all identified attributes and levels tested in the pilot is available in the Additional file 1: Table A1.

Some of the attributes, such as ‘who recommended the product’ and ‘where it is available’, were arranged (formatted) in a number of boxes underneath the initial health scenario description and above the product label. The remaining attributes, apart from price, were displayed as part of a product label, designed to be as realistic as possible and to group related attributes. Price was displayed under the labels, to represent how items are usually displayed on shop shelves. An example scenario is available in the Additional file 1: Table A2. Choice scenarios were uploaded as an online survey. Participants were asked to complete the online survey on specialized computers with eye-tracking capabilities as their first task. No specific training materials were provided to participants apart from a general introduction and a practice DCE choice set (using a transport scenario) and no prior mention of the traffic light or regulatory statements was made before the survey commenced.

### Measurement of attribute non-attendance (ANA)

Eye-tracking technology has evolved rapidly in recent years. Earlier prototypes required participants to wear bulky headwear and/or electrodes and stay in relatively uncomfortable positions for periods of time. Newer eye-trackers can be installed into regular-looking desktop computers and do not require the use of additional external hardware. For the present study, there was no requirement for headwear or electrodes and, apart from completion of a short calibration of each individual’s eyes to the screen (about 30 s) and being asked to remain as still as possible during the survey to maximise the likelihood of being detected by the eye-tracker, participants should have remained relatively unaware that they are working on anything other than a regular computer. Informed consent was obtained from all participants to use the eye-tracking technology. Here we used a Tobii T120 eye-tracker and associated software (Studio Version 2.3.2.0) to formulate the raw data which was then exported and analysed in Stata 13 statistical software [40]. The eye-tracking data so obtained consists of fixations (unique observations for each time a participant focuses or fixates on anything within the calibrated screen) and saccades and allowed identification of area of fixations, duration of fixations and order of fixations. Data for pupil dilation was also available but not made use of in this analysis.

Using the specialised Tobii software, we can build a matrix of “areas of interest” (AOI) overlaying the image for each choice set. Each AOI represents one cell and here the cells of interest are alternative-specific attributes. An example of an AOI coded choice set is provided in the Additional file 1: Figure A5. The software can then calculate a number of metrics for each AOI including the number of times each attribute was visited, how long each ‘fixation’^2^ (look) lasted and the size of the pupil. Given the large amount of data available, we limit our analysis here to the number of times an attribute was visited. From this we can calculate the inverse – whether the attribute was fixated at all during the choice set. As the level of an attribute can only influence attendance to an attribute if that attribute is first fixated, here we leave aside attribute levels as predictors of ANA.

### Statistical analysis

(i) *Description of the existence, extent and variation of attribute non-attendance (ANA) across questions and attributes:* We summarise the eye-tracking data to show the extent of attendance to each attribute across different questions and for questions with different levels of complexity (number of attributes). We present results for whether ANA occurs across both alternatives (CAM and conventional), before considering whether ANA occurs for each alternative taken individually. The ANA data is then disaggregated to describe ANA by attribute.
(ii) *Determination of the most likely contributors to ANA:* Following Lagarde [25], we hypothesise that complexity has an independent and direct effect on ANA (increased complexity is associated with increased ANA). To test this hypothesis, we regress ANA on complexity while controlling for other characteristics of the decision problem (condition and direction^3^), context (time pressure^4^), and respondent characteristics. We estimate the effect of complexity on attribute non-attendance using both fixed and random effects panel regressions. Equation (1) specifies the model:
  1 $$ {\mathrm{ANA}}_{\mathrm{i}\mathrm{j}}={\upalpha}_{\mathrm{i}}+\updelta {\mathrm{complexity}}_{\mathrm{i}\mathrm{j}}+\uptau {\mathrm{condition}}_{\mathrm{i}\mathrm{j}}+\upeta \mathrm{time}\_{\mathrm{pressure}}_{\mathrm{i}}+\upgamma {\mathrm{direction}}_{\mathrm{i}}+\upomega {\mathrm{W}}_{\mathrm{i}}+{\upvarepsilon}_{\mathrm{i}\mathrm{j}} $$
  where ANA_ij_ (attribute non-attendance) is the number of attributes with zero fixations for participant *i* in choice-set *j*; α_*i*_ captures individual-specific fixed/random effects controlling for observed and unobserved respondent characteristics; complexity_j_ is the number of attributes present in choice-set *t*; condition_j_ is a dummy indicator coded as 1 if choice-set *j* relates to the joint pain scenario (and 0 for the insomnia scenario)*;* time_pressure is a dummy indicator of whether the appointment time was late (after 5.30 pm)^5^; direction_i_ is a dummy indicator of whether the participant received choice-sets ordered in increasing (forward) or decreasing (reverse) complexity; W_i_ is the matrix of respondent characteristics; and *ε*_*ij*_ is an idiosyncratic error. The intention here is not to estimate part-worth utilities and the parameter of primary interest is δ. Where δ is positive and significant, attribute non-attendance increases with complexity (as hypothesised). We also include complexity as a quadratic term to allow a non-linear relationship between ANA and complexity.
  Included in the matrix of respondent characteristics are dummy variables for gender; a continuous measure for age (and age squared to allow for non-linear effects); a dummy variable coded 1 for education levels below university level^6^; and a dummy variable coded 1 for post-graduate students.^7^ Also included is a dummy variable indicating if the participant reported using different CM products in the previous 12 months to account for prior experience and to proxy for *a priori* preferences. Three variables are included:
  i. vitamin (self-selected) = taken a vitamin, mineral or herbal supplement not prescribed by a medical doctor in the past 12 months;
  ii. vitamin (prescribed) = taken a vitamin, mineral or herbal supplement prescribed by a medical doctor in the past 12 months;
  iii. other CAM = used other complementary and alternative medicine products or therapies (here it includes Western herbal medicines; Chinese medicines; acupuncture or indigenous or traditional folk therapies)
  We hypothesised that participants’ *a priori* preferences may make them more inclined towards choosing particular alternatives, and as the alternatives here are labelled (that is, they are specified to be ‘*conventional*’ and ‘*complementary*’ medicines rather than a generic option of ‘*Medicine A’* versus ‘*Medicine B’*), then we may also expect ANA to vary between alternatives, as well as between attributes. To account for this potential labelling effect, we also run the regression specified in Equation (1), but with ANA now ‘alternative specific’ – that is, the dependent variable is now the number of attributes not attended to within an alternative, rather than across all alternatives. This is expressed in equations (2) and (3) below:
  2 $$ \mathrm{ANA}\_{\mathrm{conv}}_{\mathrm{i}\mathrm{j}}={\upalpha}_{\mathrm{i}}+\updelta {\mathrm{complexity}}_{\mathrm{j}}+\uptau {\mathrm{condition}}_{\mathrm{j}}+\upeta \mathrm{time}\_{\mathrm{pressure}}_{\mathrm{i}}+\upgamma {\mathrm{direction}}_{\mathrm{i}}+\upomega {\mathrm{W}}_{\mathrm{i}}+{\upvarepsilon}_{\mathrm{i}\mathrm{j}} $$
  3 $$ \mathrm{ANA}\_{\mathrm{CM}}_{\mathrm{i}\mathrm{j}}={\upalpha}_{\mathrm{i}}+\updelta {\mathrm{complexity}}_{\mathrm{j}}+\uptau {\mathrm{condition}}_{\mathrm{j}}+\upeta \mathrm{time}\_{\mathrm{pressure}}_{\mathrm{i}}+\upgamma {\mathrm{direction}}_{\mathrm{i}}+\upomega {\mathrm{W}}_{\mathrm{i}}+{\upvarepsilon}_{\mathrm{i}\mathrm{j}} $$
  Definitions of explanatory variables remain consistent with equation (1).
(iii) *Consistency with which decision rules are applied:* Finally, we also test a previous assumption made by others investigating ANA [41] whereby participants are consistent with regard to which attributes they consider across choice sets (and by implication, which to ignore). To do this, we construct a measure of ‘consistency’ of individual i, detailed in Equation (4):
  4 $$ {\mathrm{consistency}}_{\mathrm{i}}=\mathrm{mean}{\left({\mathrm{s}}_{\mathrm{i}\mathrm{j}}-{\mathrm{S}}_{\mathrm{i}}\right)}^2 $$

where *s* is the proportion of attributes attended to in choice set *j* by individual *i* and *S*_*i*_ is the mean of *s* for individual *i*. Here, a higher value indicates less consistency across choice sets and more deviation in terms of the number of available attributes attended/not attended to. We then regress consistency as the dependent variable with the same set of explanatory variables detailed in equations 1, 2 and 3, with the exclusion of complexity and condition (which are invariant when considering consistency across choice-sets), as detailed in Equation (5) below:

5 $$ {\mathrm{consistency}}_{\mathrm{i}}={\upalpha}_{\mathrm{i}}+\upeta \mathrm{time}\_{\mathrm{pressure}}_{\mathrm{i}}+\upgamma {\mathrm{direction}}_{\mathrm{i}}+\upomega {\mathrm{W}}_{\mathrm{i}}+{\upvarepsilon}_{\mathrm{i}\mathrm{j}} $$

## Results

Thirty-nine participants completed the survey using the eye-tracking technology. However, the quality of eye-tracking data was insufficient in the case of seven participants, and their data is excluded in this analysis.^8^ Table 1 details the participant characteristics. The majority of participants are female (75 %), highly educated and in higher income groups. The majority (75 %) also report having taken a self-selected vitamin, mineral or herbal product in the previous 12 months.

**Table 1:** Summary of participant demographics (*N* = 32)

Female		24/32 (75 %)
Age	mean	37.4 years
	median	32 years
	range	20–70 years
Born in Australia		17/32 (53 %)
Language spoken at home	English	28/32 (88 %)
Government concession card^a^		10/32 (31 %)
Highest level of education	High school^b^ or vocational training	5/32 (16 %)
*(Higher than 100 % due to rounding)*	Undergraduate degree	15/32 (47 %)
	Postgraduate degree	12/32 (38 %)
Full-time student		5/32 16 %
Current household income^c^		
*(Higher than 100 % due to rounding)*	<$50,000	7/32 (22 %)
	$50,000- < $100,000	13/32 (41 %)
	$100,000+	12/32 (38 %)
Used vitamin last 12 months - self^d^	yes	24/32 (75 %)
Used vitamin last 12 months - dr^e^	yes	7/25 (22 %)
Used other CAM last 12 months^f^	yes	18/32 (56 %)

^a^Indicates the individual is eligible for low-income government assistance

^b^Year 11 or 12 in the Australian system (final years) – no one reported a lower level

^c^Australian dollars, 2011 (before tax)

^d^vitamin (self-selected) = taken a vitamin, mineral or herbal supplement not prescribed by a medical doctor in the past 12 months

^e^vitamin (prescribed) = taken a vitamin, mineral or herbal supplement prescribed by a medical doctor in the past 12 months

^f^other CAM = used other complementary and alternative medicine products or therapies (here it includes Western herbal medicine; Chinese medicine; CAM practitioners, or indigenous or traditional folk therapies)

We summarise attribute attendance by question in Table 2, first across the two alternatives combined and then for each alternative separately. For example, in the first line of Table 2 (for question 1) it can be seen that 32 participants (100 %) attended to all attributes in at least one of the alternatives but not all participants attended to every attribute in every alternative. 28 participants (88 %) attended to all attributes in the conventional medicine alternative and 29 participants (91 %) attended to all attributes in the conventional medicine alternative. It can be seen that attendance is relatively high for the first four questions, but drops from 100 % (all attributes attended to when considering combined alternatives) in question 1 down to 50 % in question 8. A similar pattern can be seen when considering each alternative separately; with the proportion declining as we move from question 1 to 8.

**Table 2:** Number of participants who attended to every attribute for both conventional & CM alternatives combined, and each alternative alone (*N* = 32)

Question	Number attributes	Health condition	Alts combined^a^ # participants (%)	Conv alternative # participants (%)	CM alternative # participants (%)
1	3	joint	32 (100)	28 (88)	29 (91)
2	3	insomnia	28 (88)	24 (75)	20 (63)
3	4	joint	32 (100)	26 (81)	24 (75)
4	4	insomnia	25 (78)	24 (75)	18 (56)
5	5	joint	20 (63)	13 (41)	14 (44)
6	6	insomnia	18 (56)	13 (41)	12 (38)
7	8	joint	17 (53)	13 (41)	12 (38)
8	8	insomnia	16 (50)	15 (47)	13 (41)

Abbreviations: *Alts* alternatives, *conv* conventional, *CM* complementary medicine, *#* number

^a^For a participant to have attended to an attribute, they had to have one or more fixations on that attribute, irrespective of whether they looked at the levels of the attribute in both choices

Note: The ‘neither of these’ option did not have any attributes specified and is excluded from this analysis

Across all participants, the mean number of attributes not attended to across all choice sets is 0.45 (sd 0.93, skewness 2.50, kutosis 9.61). For the conventional alternative the mean is 0.74 (sd 1.18, skewness 1.87, kurtosis 6.39) and for the CM alternative 0.75 (sd 1.12, skewness 1.82, kurtosis 6.04). The paired *t*-test for the mean difference of the two alternatives is significant (*p* = 0.05).

**Figure 1:**
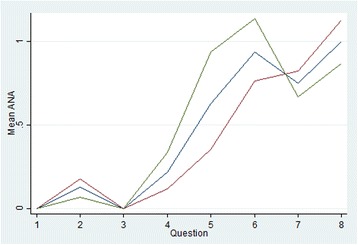
Mean attribute non-attendance by question order

**Figure 2:**
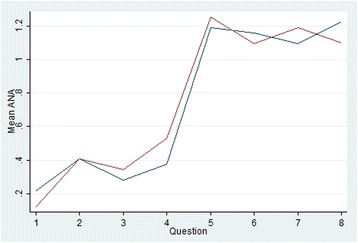
Mean conventional & Cm attribute non-attendance

**Figure 3:**
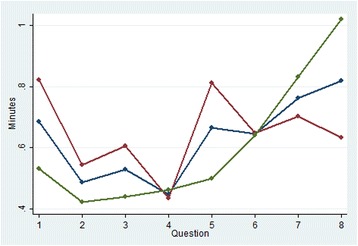
Mean time spent on each question (minutes)

The effect of viewing the questions in forward (increasing complexity) compared to reverse order is shown in Figure 1. Mean ANA is zero for questions 1 and 3 and is lower in all questions framed by the ‘joint’ scenario as compared with the corresponding ‘insomnia’ question (that is, mean ANA is less in Q1 cf Q2, Q3 cf Q4, Q5 cf Q6 and Q7 cf Q8 in the forward order and the contrary is true for the reverse order). There is slightly less ANA at question 8 by those participants who completed the survey in reverse order, however for questions 3 to 6 there is higher mean ANA for reverse order participants. In general, there is higher ANA for the questions with more attributes, irrespective of the order in which the survey was seen. Mean ANA by alternative is shown in Figure 2. Both figures show a relatively large ANA increase/drop between questions 4 and 5 (or, for reverse order, between questions 5 and 4) which is where the product labels appear/disappear for the first time, greatly increasing/decreasing the amount of information to be considered. The trends in ANA across questions for both alternatives are similar. The mean time taken to answer each choice set is shown in Figure 3 and shows that, on average, more time was spent on answering question 1 if the survey was shown in forward order, and more time on question 8 if the survey was seen in reverse order. The total curve (forward and reverse order curves combined) is broadly u-shaped, with the time taken dropping steeply if the survey is seen in forward order (from question 1 to 2) or in reverse order (from question 8 to 7).

We then look to see if there are particular attributes which are more prone to ANA than others and this is presented in Table 3. Notably, price was missed by just over 16 % of participants on average for the 5 questions in which it was available, a phenomenon that has been found by others [25] and a concern for willingness-to-pay estimates from DCEs. Other attributes that appeared more likely to be missed included where the product was available (by up to 19 % of participants) and the caution and warnings on the labels (by up to 22 and 31 % of participants, respectively). The traffic light was missed by 15 and 22 % of participants in question 7 and 8 respectively.

**Table 3:** Eye-tracking results – percent participants who did not attend to each attribute, disaggregated by within alternative non-attendance^a^

	Question number							
	1	2	3	4	5	6	7	8
Attribute								
Recommendation	0	3 %	0	0	3 %	6 %	0	0^c^
Recommendation - conv	3 %	6 %	3 %	0	9 %	9 %	6 %	10 %
Recommendation - CM	6 %	16 %	13 %	16 %	19 %	19 %	19 %	19 %
Side effects	0	3 %	0	0	13 %	3 %	6 %	6 %
Side effects - conv	9 %	13 %	6 %	6 %	16 %	13 %	13 %	16 %
Side effects - CM	3 %	9 %	6 %	16 %	28 %	16 %	19 %	16 %
Where available	0	6 %	0	9 %	19 %	9 %	3 %	16 %
Where available - conv	9 %	22 %	9 %	16 %	28 %	19 %	16 %	26 %
Where available - CM	3 %	16 %	6 %	9 %	31 %	19 %	13 %	23 %
Price	NA	NA	0	13 %	16 %	22 %	13 %	19 %
Price - conv	NA	NA	9 %	16 %	34 %	44 %	41 %	39 %
Price - CM	NA	NA	9 %	13 %	34 %	34 %	28 %	26 %
*Dosage* ^*b*^	NA	NA	NA	NA	0	0	6 %	6 %
*Dosage* ^*b*^ *- conv*	NA	NA	NA	NA	9 %	13 %	6 %	6 %
*Dosage* ^*b*^ *- CM*	NA	NA	NA	NA	3 %	3 %	9 %	10 %
Caution	NA	NA	NA	NA	13 %	22 %	3 %	10 %
Caution - conv	NA	NA	NA	NA	31 %	NA	16 %	NA
Caution - CM	NA	NA	NA	NA	13 %	22 %	9 %	10 %
Warning	NA	NA	NA	NA	NA	31 %	19 %	10 %
Warning - conv	NA	NA	NA	NA	NA	31 %	19 %	10 %
Warning - CM	NA	NA	NA	NA	NA	NA	NA	NA
Traffic light	NA	NA	NA	NA	NA	NA	16 %	23 %
Traffic light - conv	NA	NA	NA	NA	NA	NA	NA	23 %
Traffic light - CM	NA	NA	NA	NA	NA	NA	16 %	NA
Regulation – CM (only)	NA	NA	NA	NA	NA	NA	16 %	16 %

*NA* not applicable – the attribute did not appear in the particular question

*Conv* conventional medicine, *CM* complementary medicine

^a^The corresponding questions, whether seen in forward or reverse order, are combined here and presented as if the forward order has been seen by the participant (ie. question 1 data in the forward order and question 8 data in the reverse order has been aggregated)

^b^Dosage was considered to be a fixed attribute (the levels did not change) – it was included for realism

^c^Denominator is 31 participants in question 8 due to missing eye-tracking data for participant 124

Results from the main regressions are presented in Table 4. Our main interest is the relationship between ANA and complexity, which shows a positive and significant main effect for models 1–4, with a negative and significant quadratic term (that is, ANA is increasing with complexity but at a diminishing rate over the number of attributes we tested here). The fixed and random effects models (models 1 and 2, respectively) provided similar estimates and tests for the appropriateness of using the random effects model did not reject the null that results are consistent (see the footnote to Table 4 for details). We also re-run the model after centring the mean of complexity at zero and although the beta coefficients on complexity differ, the sign and significance are unchanged.

**Table 4:** Summary of main results from eye-tracking regression of attribute non-attendance (ANA)

	(1) Number ANA, fe			(2) Number ANA, re			(3) Number ANA (conv), re			(4) Number ANA (CM), re			(5) Consistency^a^		
	b	se	p	b	se	p	b	se	p	b	se	p	b	se	p
complexity	0.578^b^	0.210	0.006	0.578^c^	0.230	0.012	0.749^b^	0.247	0.002	0.863^b^	0.309	0.005			
complexity2	−0.036^d^	0.019	0.057	−0.036^d^	0.020	0.081	−0.050^c^	0.024	0.037	−0.061^c^	0.027	0.027			
joint	−0.182^d^	0.097	0.062	−0.182^d^	0.097	0.060	−0.046	0.132	0.728	−0.013	0.094	0.887			
late appointment				0.481^d^	0.275	0.080	0.438	0.308	0.155	0.680^d^	0.412	0.098	0.019	0.011	0.112
forward order				0.005	0.140	0.973	0.154	0.210	0.462	0.029	0.228	0.897	−0.006	0.007	0.412
female				0.104	0.144	0.471	0.017	0.187	0.927	0.234	0.188	0.214	0.003	0.007	0.655
age				0.001	0.037	0.982	−0.022	0.046	0.630	−0.015	0.053	0.775	−0.004^c^	0.002	0.038
age2				0.000	0.000	0.577	0.001	0.000	0.134	0.000	0.001	0.482	0.000^c^	0.000	0.017
<uni education				0.218	0.200	0.275	0.431^d^	0.223	0.053	0.010	0.255	0.970	−0.002	0.010	0.817
student				−0.019	0.156	0.905	0.024	0.187	0.896	−0.125	0.237	0.598	−0.009	0.009	0.346
vit (self-selected)				−0.250	0.176	0.154	−0.183	0.239	0.444	−0.328	0.277	0.236	−0.011	0.008	0.190
vit (prescribed)				0.116	0.171	0.496	−0.189	0.192	0.325	0.456^c^	0.218	0.036	0.014	0.008	0.104
other CAM				0.027	0.145	0.853	0.088	0.154	0.568	−0.022	0.188	0.906	0.007	0.006	0.312
Constant	−1.348^c^	0.532	0.012	−1.737^c^	0.765	0.023	−1.975^d^	1.055	0.061	−1.970^d^	1.083	0.069	0.087^c^	0.041	0.046
Observations^e^	255			255			255			255			32		
*R* ^2^	.210			.276			.356			.245			.612		

Abbreviations: *ANA* attribute non-attendance OR attributes not attended (to), *complexity2* complexity squared, *age2* age squared, *uni* university, *conv* conventional medicine, *CM* complementary medicine, *vit (self-selected)* taken a vitamin, mineral or herbal supplement not prescribed by a medical doctor in the past 12 months; *vit (prescribed)* taken a vitamin, mineral or herbal supplement prescribed by a medical doctor in the past 12 months; *other CAM* used other complementary and alternative medicine products or therapies (here it includes Western herbal medicine; Chinese medicine; CAM practitioners, or indigenous or traditional folk therapies)

^a^As measured by the mean(s_ij_-S)^2^ where s is the proportion of attributes attended to in choice set *j* by individual *i* and S_i_ = mean (s) for individual *i* [whereby a higher value indicates less consistency and more deviation in terms of attribute non-attendance]

^b^, ^c^,^d^ indicates significance at the 1, 5 and 10 % levels respectively

^e^Observations are based on data from 32 participants, however, eye-tracking data is absent for question 8 for one participant (124)

We test for the appropriateness of using a random effects model using a robust Hausman test using a Wald test and cluster-robust standard errors (Wooldrige, 2002) after excluding participant 124 for whom there is missing eye-tracking data for question 8 (the scalar theta cannot be calculated for an unbalanced panel). The null hypothesis assumes that individual effects are random and both fixed and random effect estimators are consistent. The test does not reject the null (*p* = 0.652). We also perform an over-identification test with the null-hypothesis (participant 124 included) that the group means are uncorrelated with the idiosyncratic error term. The test does not reject the null (*p* = 0.911). From this we conclude that the random effects estimator results are appropriate

ANA was less likely for the joint scenario and more likely for participants who had a late appointment (both significant at the 10 % level in model 2), although the effect of the late appointment was not robust to different cut-off times. The order in which the survey was completed was not found to be associated with ANA. Some variation was shown in the relationship between socio-demographic variables and alternative-specific ANA: lower levels of education were associated with higher ANA in the conventional medicine alternative and those who had taken a vitamin prescribed by a medical doctor in the previous 12 months were more likely to miss attributes in the CM alternative.

The mean for the measure of consistency across the sample was 0.016 (sd 0.020, skewness 1.76, kutosis 5.84), with 10 participants having a mean of zero (that is, they were entirely consistent in terms of how many attributes were missed across all choice sets). In terms of the consistency regression (model 5), younger age was associated with greater consistency, although as shown by the positive and significant coefficient on the corresponding quadratic term, this effect decreases as age increases.

## Discussion and conclusions

This paper adds to the growing literature regarding attribute non-attendance in DCEs and to our knowledge is the first to explicitly focus on the relationship between complexity and ANA for decicions regarding health service utilisation Our results show there is a strong positive and statistically significant relationship between ANA and complexity and that this relationship is robust to a number of different model specifications. Importantly, we find that complexity is the strongest predictor of ANA when other possible influences, such as time pressure, ordering effects, survey specific effects and socio-demographic variables (including proxies for prior experience of the decision problem) are considered. We also find that ANA, as well as the consistency with which attribute attendance is applied across choice sets, does show some evidence of heterogeneity across different socioeconomic variables, specifically for education and age. Like others, we do find considerable departure from the assumptions underpinning RUT which assumes consumers maximise their utility based on all available information [25, 30]. Similar to Balcombe [30], we found that full attendance to all attributes across all choice sets is unusual, however, ANA was significantly less present for choice sets with fewer attributes.^9^

The interpretation of this finding should be taken within the context of this particular study. In general participants reported being engaged with the survey and although many stated that the choice sets with more information took longer to process, the information itself was not difficult to understand. Most also reported that they thought all attributes were potentially relevant to their decision and there were no recommendations to remove particular attributes (only to change one of the levels of one of the attributes).

What has yet to be clearly determined in the literature is whether, and the extent to which, utility functions should be adjusted for ANA. The present study was conducted alongside the pilot for a DCE and varied the number of attributes across choice sets to identify the effect of complexity on ANA. As a consequence, we observed limited variation across attribute levels for some attributes and could not account for the effect of all attributes when estimating utility functions. Lagarde [25] found that whilst willingness-to-pay estimates were sensitive to ANA, the behavioural prediction of DCE models was not affected by ANA. One explanation for this may be that consumers are so accustomed to using heuristics or decision rules in complex or uncertain situations that they are well practised in seeking out information that will be useful to them in their final decision (in essence, conferring zero utility for any attributes superfluous to their needs). Thus, reading attribute and alternative labels may be sufficient for some consumers to decide if the subsequent information available is worthwhile attending to or not.

We did, however, find evidence that ANA differed across alternatives, although the mean effect was shown to be small. While we cannot rule out here that this effect may also represent left-right logographical ordering, differences in socio-demographic determinants of alternative-specific ANA such as prior use of a prescribed vitamin are perhaps more consistent with a CM-CAM effect than a left-right effect. In any event, the effect of alternative-specific ANA on utility functions, as compared to ‘total’ ANA for a given attribute is worthy of further consideration (regardless of whether it represents a CM-CAM or left-right effect). Alternative-specific ANA may also offer additional insights into the decision processing strategy used by participants during DCEs.

Other results were also interesting. As seen in Figure 1, ANA was consistently lower for the questions framed by the ‘joint’ scenario (questions 1, 3, 5 & 7 in the forward order) compared with the corresponding ‘insomnia’ questions. This may indicate a framing effect, whereby participants were more likely to not attend to attributes in the insomnia questions, perhaps due to strongly formed opinions as to how each ailment ‘should’ be treated (prior experience) or strong preferences for natural or conventional medicines in specific contexts. Aside from the framing effect, the general trend for more ANA in questions with more attributes supports the notion that increased complexity is linked with more ANA irrespective of the order in which questions were seen. The time taken to answer each question (Fig. 3) broadly displays a ‘U’ shape for the combined forward and reverse order surveys (total sample line), perhaps suggesting a learning effect which means the time taken decreases to a point before fatigue starts to increase. However, the forward curve consistently shows longer times taken for questions 1, 3, 5 & 7 (joint scenario questions) compared with the corresponding insomnia questions (which interestingly corresponds to lower ANA for the joint questions compared with the insomnia questions in Fig. 1). This is not seen for the reverse order curve which shows consistently decreasing times taken for questions 8 to 2, increasing slightly again for the final question 1. It is not apparent why a framing effect might be present only in the forward order survey and this is worthy of further consideration.

The finding that ‘consistency’ with regard to the number of attributes attended to across choice sets decreased with age may be potentially explained by a decrease in cognitive function over time, although this cannot be tested here. Results are not consistent with the assumption made by Hole [41] that the decision of which attribute/s to consider is stable across choice sets and are instead more supportive of the notion that this varies over choice sets, as suggested by others [26].

This study also has some important implications for the design of DCEs measuring health and health-care preferences more generally. This study, which also acted as a pilot for a larger DCE, highlights the design complexity of some of the scenarios encountered by health researchers and raises further questions about how the qualitative properties of the survey, such as the description of attributes and levels, presentation of choice sets and clarity of instructions may impact on ANA. When combined with findings regarding the effect of ANA on utility estimates, our findings regarding the effect of complexity on ANA should permit identification of the point at which *adding* complexity (and realism) to DCE choice scenarios becomes self-defeating.

One of the obvious limitations of this analysis is the small and unrepresentative sample size. Despite avoiding an entire undergraduate student population, the recruited sample remained better educated and from higher socioeconomic circumstances than the general population. The majority (75 %) of participants reported self-selection of a vitamin, mineral or herbal product in the previous 12 months which is higher than reports in the literature for Australian populations [42]. For this presumably less ‘boundedly’ rational sample, we might expect additional information to evoke fewer changes in information processing than for the general population [43]. Therefore, our results are likely to underestimate ANA in the general population. Additionally, we only tested complexity over a range of 3–8 attributes, which is the upper limit of attributes reported to be routinely included in DCEs in the health setting [2]. It must be remembered that some attributes are only seen in two questions (for example, the regulatory statements and traffic light logos are only seen in questions 7 & 8). Thus, caution should be exercised in drawing conclusions regarding the effect of additional attributes in other DCE studies. Further, we did not set out to test the effect of the location (page orientation) of attributes as it relates to ANA, whereby there may be a systematic difference due to orientation alone (eg. the bottom of the page may be more prone to ANA).

The rapid advancements in eye-tracking technology over recent years have meant that this technology is likely to be used more extensively to investigate questions of information processing across a range of disciplines, including in health economics. Alongside this, methodological questions also need to be answered regarding the use of other available metrics (fixations, saccades, pupil dilation), the definitions applied (for example, ANA) and how these may be linked to neurological process to provide greater insight into decision-making processes. Recent progress on this front suggests that the full potential of combining eye-tracking data with more familiar qualitative and quantitative data is yet to be realised.

## Additional file

Additional file 1: **Supplementary material.** (DOCX 592 kb)

## Abbreviations

ANA: attribute non-attendance
AOI: area of interest
CAM: complementary and alternative medicine
CM: complementary medicine DCE – discrete choice experiment
RUT: random utility theory
TGA: therapeutic good administration

## Footnotes

1 This study was also used to pilot test the attributes and levels of the DCE for use in a subsequent study using a larger sample size.

2 The eye-tracker collects raw data every 16.7 milliseconds and assigns to each data point a location. A fixation filter is then applied to determine if each data point is a ‘fixation’ or ‘saccade’ (for two points to be considered as part of the same fixation, the distance between two data points must be below a minimal threshold). We used the default ‘ClearView’ settings for the I-VT (Velocity Threshold Identification) fixation filter [Tobii Studio 2.X, Release 2.2, User Manual (2010). http://www.tobii.com/].

3 Dummy variables indicating (i) whether the choice set relates to the joint pain scenario or to the insomnia scenario and (ii) whether the survey was seen in increasing order of complexity (forward) or decreasing order of complexity (reverse) were included in the model.

4 The time taken to complete each choice set was recorded during the experiment, however, this measure is likely to reflect complexity and respondent characteristics rather than context. A suitable proxy for time pressure was therefore identified. As the appointment time for each participant varied, we reasoned that appointments later in the day were more likely to be associated with greater time pressure as changes in traffic conditions and outside work activities are more likely to be given higher consideration around this time.

5 This cut-off was chosen as it is a time when most people have finished work for the day. Only three individuals were classified as having a late appointment using this definition. The robustness of the cut-off is tested during the analysis and reported in the results section.

6 Due to the sample being drawn from a university, this variable is also likely to indicate professional (non-academic) staff status.

7 Undergraduate students were excluded from participating.

8 The eye-tracking software provides a percentage of the time over the duration of the survey for which eye-tracking data was collected. If participants did not remain still enough, for example, and data was not able to be captured for some of the time, the percentage was less than 100 %. As a general rule, we excluded participants for this analysis if their percentage tracked was 50 % or less, however, this is an overall figure which includes time spent on both the DCE choice sets and introduction/demographics sections, and it was relaxed in the case of six participants where it was deemed there was sufficient data capture during the DCE section for them to be included.

9 It is also important to note that Balcombe used a different definition of ANA whereby meeting or exceeding the threshold of two fixations per attribute defined attendance, whereas we used the stricter definition of zero fixations to define non-attendance.

## References

[1] de Bekker-Grob EW, Ryan M, Gerard K (2012). Discrete choice experiments in health economics: a review of the literature. Health Econ.

[2] Johnson FR (2013). Constructing experimental designs for discrete-choice experiments: report of the ISPOR conjoint analysis experimental design good research practices task force. Value Health.

[3] Lancsar E, Louviere J (2008). Conducting discrete choice experiments to inform Healthcare decision making. Pharmacoeconomics.

[4] Clark MD, Determann D, Petrou S, Moro D, de Bekker-Grob EW (2014). Discrete choice experiments in health economics: a review of the literature. Pharmacoeconomics.

[5] McFadden D (1973). Conditional Logit Analysis of Qualitative Choice Behaviour.

[6] Hanemann WM (1984). Welfare evaluations in contingent valuation experiments with discrete responses. Am J Agric Econ.

[7] Lancsar E, Louviere J (2006). Deleting ‘irrational’ responses from discrete choice experiments: a case of investigating or imposing preferences?. Health Econ.

[8] Ryan M, Bate A (2001). Testing the assumptions of rationality, continuity and symmetry when applying discrete choice experiments in health care. Appl Econ Lett.

[9] Simon HA (1955). A behavioural model of rational choice. Q J Econ.

[10] Simon HA, McGuire CB, Radner R (1972). Theories of bounded rationality. Decision and Organisation.

[11] Depalma A, Myers GM, Papageorgiou YY (1994). Rational choice under an imperfect ability to choose. Am Econ Rev.

[12] Simon HA (1990). Invariants of human-behavior. Annu Rev Psychol.

[13] Gigerenzer G, Todd PM (1999). Simple Heuristics That Make us Smart.

[14] Tversky A, Kahneman D (1974). Judgment under uncertainty: heuristics and biases. Science.

[15] Kooreman P, Prast H (2010). What does behavioral economics mean for policy? challenges to savings and health policies in the Netherlands. De Economist.

[16] Hensher DA (2006). How do respondents process stated choice experiments? Attribute consideration under varying information load. J Appl Econ.

[17] Hensher D, Rose J, Greene W (2012). Inferring attribute non-attendance from stated choice data: implications for willingness to pay estimates and a warning for stated choice experiment design. Transportation.

[18] Rose JM (2012). Attribute exclusion strategies in airline choice: accounting for exogenous information on decision maker processing strategies in models of discrete choice. Transportmetrica.

[19] Hess S, Rose JM, Polak JW (2010). Non-trading, lexicographic and inconsistent behaviour in stated choice data. Transport Res Part D: Transport Environ.

[20] Ryan M, Watson V, Entwistle V (2009). Rationalising the ‘irrational’: a think aloud study of discrete choice experiment responses. Health Econ.

[21] Coast J (2012). Using qualitative methods for attribute development for discrete choice experiments: issues and recommendations. Health Econ.

[22] Campbell D, Hensher DA, Scarpa R (2011). Non-attendance to attributes in environmental choice analysis: a latent class specification. J Environ Plan Manag.

[23] Hole AR (2011). A discrete choice model with endogenous attribute attendance. Econ Lett.

[24] Scarpa R, Thiene M, Hensher DA (2010). Monitoring choice task attribute attendance in nonmarket valuation of multiple park management services: does It matter?. Land Econ.

[25] Lagarde M (2013). Investigating attribute non-attendance and its consequences in choice experiments with latent class models. Health Econ.

[26] Hess S, Hensher DA (2010). Using conditioning on observed choices to retrieve individual-specific attribute processing strategies. Transport Res Part B-Methodol.

[27] Rayner K (1978). Eye movements in reading and information processing. Psychol Bull.

[28] Rasch C, Louviere JJ, Teichert T (2015). Using facial EMG and eye tracking to study integral affect in discrete choice experiments. Journal of Choice Modelling.

[29] Arieli A, Ben-Ami Y, Rubinstein A (2011). Tracking decision makers under uncertaint. Am Econ J Microeconomics.

[30] Balcombe, K., I. Fraser, and E. McSorley, Visual attention and attribute attendance in multi-attribute choice experiments. J Appl Econom 2014: p. n/a-n/a.

[31] Spinks J, Mortimer D (2015). The effect of traffic lights and regulatory statements on the choice between complementary and conventional medicines: results from a discrete choice experiment. Soc Sci Med.

[32] Harvey KJ (2009). A review of proposals to reform the regulation of complementary medicines. Aust Health Rev.

[33] Harvey KJ (2008). Commercialism, choice and cor sumer protection: regulation of complementary medicines in Australia - In reply. Med J Aust.

[34] Tippet G (2011). Trick or treat?. The Age.

[35] Grunert K, Wills J (2007). A review of European research on consumer response to nutrition information on food labels. Journal of Public Health.

[36] Sonnenberg L (2013). A traffic light food labeling intervention increases consumer awareness of health and healthy choices at the point-of-purchase. Prev Med.

[37] Manderson L, Canaway R, Unantenne N, Oldenburg B, Lin V, Hollingsworth B (2012). Care seeking, use of complementary therapies and self management among people with type 2 diabetes and cardiovascular disease. Aust J Herb Med.

[38] Manderson L, Oldenburg B, Lin V, Hollingsworth B, De Courten M, Canaway R (2012). Care-seeking, complementary therapy and herbal medicine use among people with type 2 diabetes and cardiovascular disease: CAMELOT Phase II, surveying for diversity. Aust J Herb Med.

[39] Spinks J, Hollingsworth B, Manderson L, Lin V, Canaway R (2013). Costs and drivers of complementary and alternative medicine (CAM) use in people with type 2 diabetes or cardiovascular disease. Europ J IntegratMed.

[40] StataCorp (2013). Stata Statistical Software: Release 13.

[41] Hole AR, Kolstad JR, Gyrd-Hansen D (2013). Inferred vs. Stated attribute non-attendance in choice experiments: A study of doctors’ prescription behaviour. J Econ Behav Organ.

[42] Spinks J, Hollingsworth B (2012). Policy implications of complementary and alternative medicine (CAM) use in Australia: data from the national health survey. J Alternat ComplementMed.

[43] Choi S, Kariv S, Müller W, Silverman D. *Who is (More) Rational?* NBER Working Paper No. 16791, 2011. Available at: http://www.nber.org/papers/w16791.pdf Accesssed 20/07/2014.

