# Supplementary material for: Lost in the crowd? Using eye-tracking to investigate the effect of complexity on attribute non-attendance in discrete choice experiments
Source: BMC Med Inform Decis Mak. 2016 Feb 3;16:14. doi: 10.1186/s12911-016-0251-1 (PMC4739384; doi:10.1186/s12911-016-0251-1)
Supplement: Supplementary file 1 — Supplementary material. (DOCX 592 kb) [file 12911_2016_251_MOESM1_ESM.docx]

**Online Appendix**

**Figure A1: Wording of the proposed statements to appear on complementary medicines**

| 1. This product has not been evaluated by Australian Health Authorities for efficacy | 1. This product has been evaluated by Australian Health Authorities for efficacy* |
| --- | --- |
| 1. Untested | 1. (No label) |

* Although this statement was not suggested in the media, we thought it appropriate to present as a more positive version of the label that was suggested.

**Figure A2: The ‘traffic-light system’ used in the pilot study as an alternative to the statements shown in Figure 1**

| **Effective for insomnia** |
| --- |
| **Interactions with medicines - moderate** |
| **Likely safe** |

**Notes:** The traffic light system was designed to describe three main aspects of the CM or conventional medicine product, namely, effectiveness; the potential for interactions with other medicines (CM and conventional); and the potential for side effects. Here, green indicates the most favourable classification; orange indicates that more care needs to be taken; and red indicates caution. It was broadly modelled on food nutrition labels. One of the key motivations of the pilot study was to test the design and comprehension of this logo. Following the pilot, this logo was updated to reflect participant comments.

**Figure A3: Example gaze plot for a participant who attended to all attributes within a question**
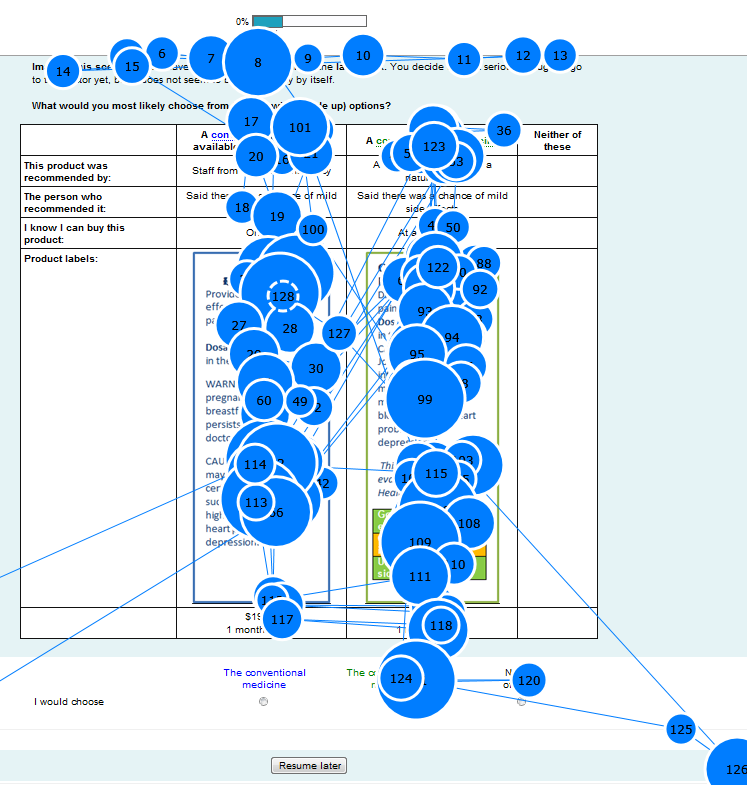


**Notes:** The Gaze Plot visualization shows the sequence and position of fixations (dots) on a static media. The size of the dots indicates the fixation duration and the numbers in the dots represent the order of the fixations.

**Figure A4: Example gaze plot for a participant who did attend to all attributes within a question**


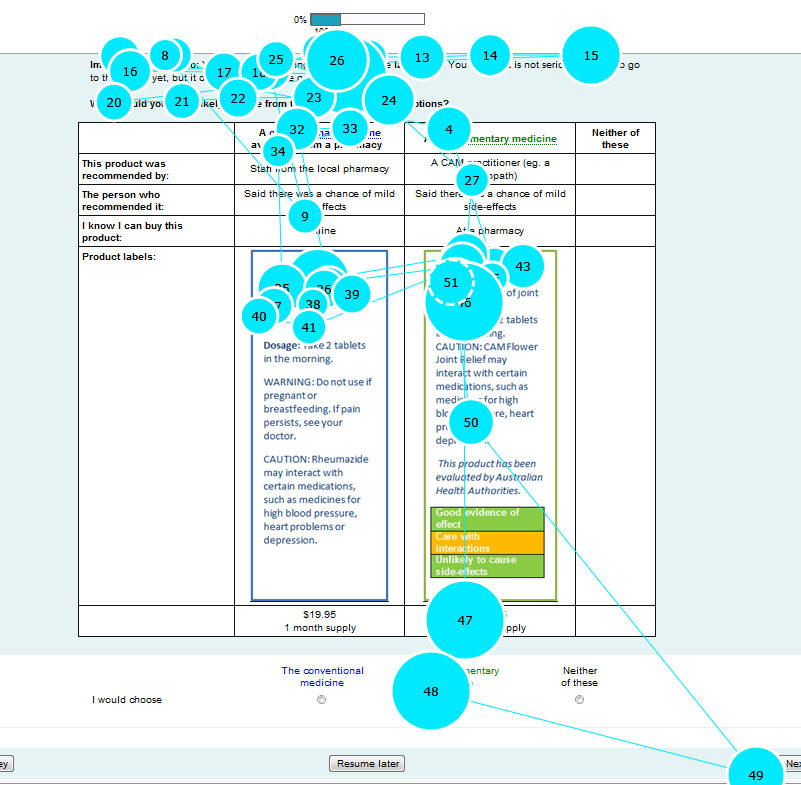


**Notes:** The Gaze Plot visualization shows the sequence and position of fixations (dots) on a static media. The size of the dots indicates the fixation duration and the numbers in the dots represent the order of the fixations.

**Table A1: Levels and attributes tested in the survey**

| **Attribute** | **Level** | **Conventional medicine** | **CAM medicine** |
| --- | --- | --- | --- |
| RECOMMENDED | | | |
| This product was recommended by: | 0 | A pharmacist | A pharmacist |
|  | 1 | A Naturopath | A Naturopath |
|  | 2 | Staff from the local pharmacy | Staff from the local pharmacy |
|  | 3 | A friend or relative or someone I know who has [Scen 1] trouble sleeping OR [Scen 2] with joint pain | A friend or relative or someone I know who has [Scen 1] trouble sleeping OR [Scen 2] with joint pain |
| SIDE EFFECTS | | | |
| The person who recommended it: | 0 | Said there was a chance of mild side-effects, like a [Scen 1] headache OR [Scen 2] constipation | Said there was a chance of mild side-effects, like a [Scen 1] headache OR [Scen 2] constipation |
|  | 1 | Didn’t mention or know anything about side-effects | Didn’t mention or know anything about side-effects |
| AVAILABLE | | | |
| I know I can buy this product: | 0 |  | From a naturopath |
|  | 1 |  | At a health food shop |
|  | 2 |  | At the supermarket |
|  | 3 | At a pharmacy | At a pharmacy |
| DOSEAGE (held constant) | | | |
|  | 0 | [Scen 1]“1 tablet one hour before bedtime” or [Scen 2] “2 tablets in the morning with food” | [Scen 1]“1 tablet one hour before bedtime” or [Scen 2] “2 tablets in the morning with food” |
| CAUTION | 0 | No caution on label | No caution on label |
|  | 1 | May interact with certain medicines, such as medicines for [Scen 1] high blood pressure, heart disease or depression OR [Scen 2] pain, anxiety or depression. | May interact with certain medicines, such as medicines for [Scen 1] high blood pressure, heart disease or depression OR [Scen 2] pain, anxiety or depression. |
| WARNING | 0 | No warning on label | No warning on label |
|  | 1 | [Scen 1] “Do not use if pregnant or breastfeeding. If pain persists, see you doctor” [Scen 2] “May cause drowsiness. Do not drive or operate heavy machinery if affected”. | [Scen 1] “Do not use if pregnant or breastfeeding. If pain persists, see you doctor” [Scen 2] “May cause drowsiness. Do not drive or operate heavy machinery if affected”. |
| TRAFFIC LIGHT | 0 | No traffic light on label | No traffic light on label |
|  | 1 | Traffic light on label (compatible with label information) | Traffic light on label (compatible with label information) |
| REGULATION | 0 | No label (held constant) | No label |
|  | 1 |  | “This product has NOT been evaluated by Australian Health Authorities for efficacy” |
|  | 2 |  | “This product HAS been evaluated by Australian Health Authorities for efficacy” |
|  | 3 |  | “Untested by Australian health authorities” |
| PRICE | 1 | $8.95 | $8.95 |
|  | 2 | $14.95 | $14.95 |
|  | 3 | $23.70 | $23.70 |
|  | 4 | $31.50 | $31.50 |

**Table A2: Example of a question (here question 7) from the survey**

**Imagine this scenario:** You have been having **joint pain** for the **last week.** You decide it is not serious enough to go to the doctor yet, but it does not seem to be going away by itself.

**What would you most likely choose from the following (made up) options?**

|  | **A conventional medicine available from a pharmacy** | **An complementary medicine** | **Neither of these** |
| --- | --- | --- | --- |
| **This product was recommended by:** | Staff from the local pharmacy | A CAM practitioner (eg. a naturopath) |  |
| **The person who recommended it:** | Said there was a chance of mild side-effects | Said there was a chance of mild side-effects |  |
| **I know I can buy this product:** | Online | At a pharmacy |  |
|  |  |  |  |
| **Product labels:** |  |  |  |

**Rheumazide**

Provides temporary effective relief of joint pain.

**Dosage**: Take 2 tablets in the morning.

WARNING: Do not use if pregnant or breastfeeding. If pain persists, see your doctor.

CAUTION: Rheumazide may interact with certain medications, such as medicines for high blood pressure, heart problems or depression.

**CAMFlower Joint Relief Formula**

Drug free relief of joint pain.

**Dosage:** Take 2 tablets in the morning.

CAUTION: CAMFlower Joint Relief may interact with certain medications, such as medicines for high blood pressure, heart problems or depression.

*This product has been evaluated by Australian Health Authorities.*

| **Good evidence of effect** |
| --- |
| **Care with interactions** |
| **Unlikely to cause side-effects** |

**$ 19.95**

**1 month supply**

**$ 19.95**

**1 month supply**

| **I would choose:** | 🞅 | 🞅 | 🞅 |
| --- | --- | --- | --- |

**Figure A5: An example choice set with overlaying Areas of Interest (AOI) used in the eye-tracking software to determine fixation metrics**

**
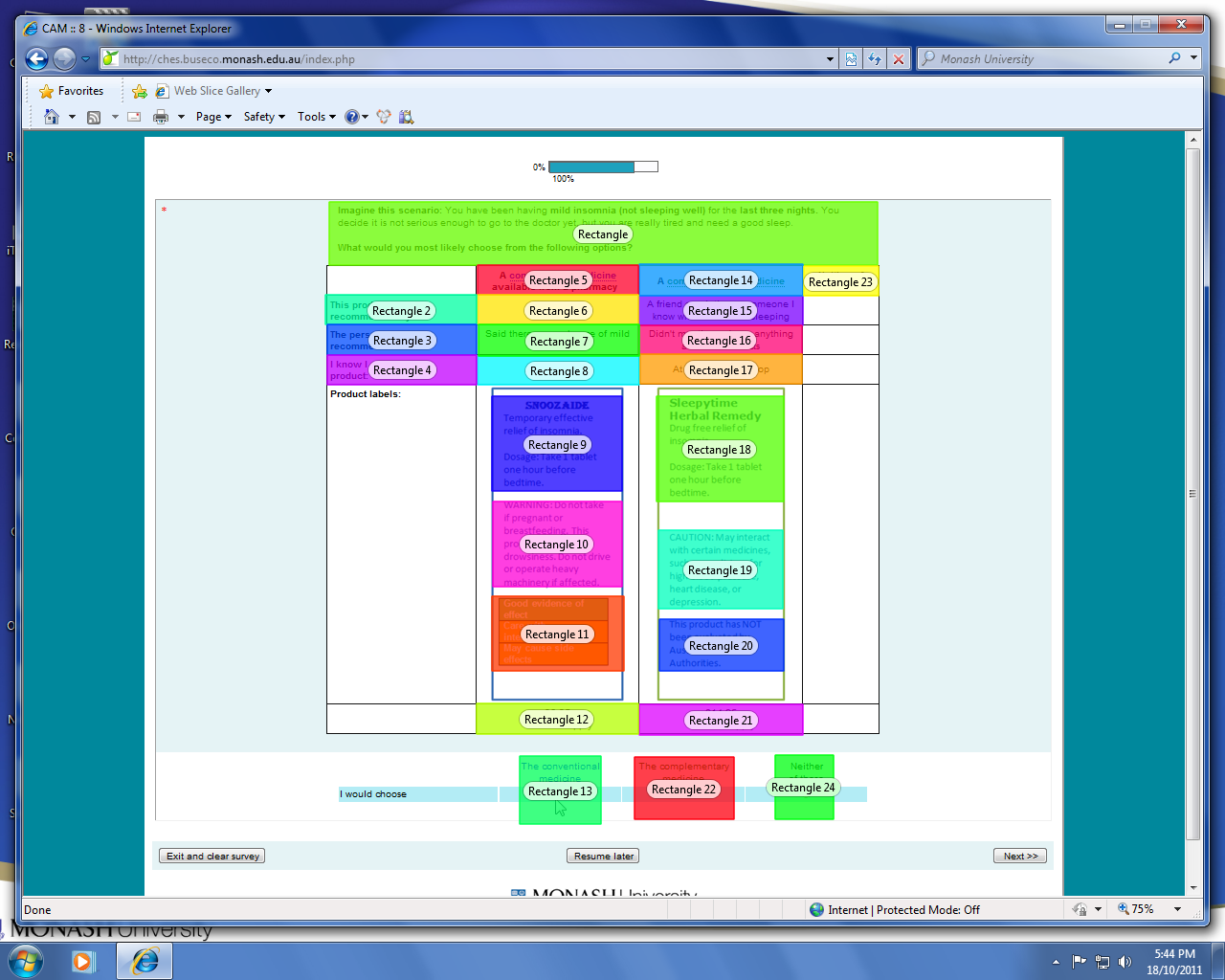
**

**Notes:** AOIs were alternative specific and overlayed on the attributes in such a way that all of the ‘cell’ was covered but fixations on empty space would not be included.
